# Abacavir use is associated with increased prothrombin conversion

**DOI:** 10.3389/fimmu.2023.1182942

**Published:** 2023-04-14

**Authors:** Qiuting Yan, Shengshi Huang, Wouter van der Heijden, Marisa Ninivaggi, Lisa van de Wijer, Romy de Laat-Kremers, Andre J. Van der Ven, Bas de Laat, Quirijn de Mast

**Affiliations:** ^1^ Department of Functional Coagulation, Synapse Research Institute, Maastricht, Netherlands; ^2^ Department of Biochemistry, Cardiovascular Research Institute Maastricht (CARIM), Maastricht University, Maastricht, Netherlands; ^3^ Department of Internal Medicine, Radboud Center for Infectious Diseases, Radboud University Medical Center, Nijmegen, Netherlands; ^4^ Department of Data Analysis and Artificial Intelligence, Synapse Research Institute, Maastricht, Netherlands

**Keywords:** HIV, abacavir, thrombin generation, thrombin dynamics, coagulation

## Abstract

There is ongoing debate as to whether abacavir (ABC) increases the risk for cardiovascular disease(CVD) in people living with HIV (PLHIV) and the mechanisms underlying this possible association. We recently showed that the use of an ABC-containing regimen was independently associated with increased thrombin generation (TG). In the present study, we aim to explore these findings further, by studying the mechanistical processes that underly the global thrombin generation test via thrombin dynamics analysis. Thrombin dynamics analysis can pinpoint the cause of increased thrombin generation associated with ABC-use either to the procoagulant prothrombin conversion pathway or the anticoagulant thrombin inactivation pathway. In this cross-sectional study, 208 virally suppressed PLHIV were included, of whom 94 were on a ABC-containing regimen, 92 on a tenofovir disoproxil fumarate (TDF)-containing regimen, and the remainder on other regimens. We used Calibrated Automated Thrombinography to measure thrombin generation and perform thrombin dynamics analysis. The total amount of prothrombin conversion, as well as the maximum rate of prothrombin conversion were significantly increased in PLHIV on an ABC containing regimen compared to other treatment regimens. The levels of pro- and anticoagulant factors were comparable, indicating that the ABC-induced changes affect the kinetics of prothrombin conversion rather than procoagulant factor levels. Moreover, Von Willebrand Factor (VWF), active VWF and VWF pro-peptide levels were significantly higher in PLHIV than controls without HIV. However, they did not differ between ABC and non-ABC treated participants.

## 1 Introduction

Combination antiretroviral therapy (cART) protects people living with HIV (PLHIV) from the progression of the HIV infection to acquired immunodeficiency syndrome (AIDS) (1). Additionally, cART protects PLHIV against non-AIDS comorbidities, such as cardiovascular diseases (CVD) (2). This increased risk of CVD is associated with the activation of inflammatory and hemostatic pathways (1). HIV-infection has been recognized as a prothrombotic condition and is associated with an increased risk of venous thromboembolism (VTE) compared to the general population (1). Furthermore, HIV-infection has been reported to increase the risk of recurrent VTE (2), myocardial infarction (3) and ischemic stroke (4). Moreover, the association of abacavir (ABC)-based treatments and an increased risk of myocardial infarction has been under debate (3–8). The mechanisms underlying this possible association remain incompletely understood.

The coagulation system can be assessed by the thrombin generation (TG) test by applying Calibrated Automated Thrombinography (CAT). The CAT assay is sensitive to small alterations in coagulation (9). An increase of TG peak height and endogenous thrombin potential (ETP) is associated with hypercoagulability and, vice versa, a low TG peak height and ETP is associated with hypocoagulability (10, 11). Higher TG peak height and ETP are known to a risk factor for first and recurrent VTE (12–15). Moreover, higher TG peak height and ETP are associated with an increased risk of mortality in the general population (16). Recently, we showed that ABC-based treatment was independently associated with an increase in TG peak height and ETP, compared to non-ABC regimens (17). Higher TG peak height and ETP have been association with an increased risk of cardiovascular diseases in the general population (18). High peak height and ETP are associated with an increased risk of stroke (19, 20) and cardiovascular mortality (21).

TG is a global hemostasis assay that integrates the pro- and anticoagulant processes that take place in clotting plasma (13). Therefore, the outcome of the TG assay is a representation of the hemostatic balance. The main processes that take place during thrombin generation are prothrombin conversion and thrombin activation (22). Subsequently, an increase in TG potential is either by an increase of prothrombin conversion, a reduction of thrombin inactivation, or a combination of both (23). Changes in prothrombin conversion and thrombin activation can be analyzed by applying thrombin dynamics methodology (22, 24, 25). Thrombin dynamics analysis quantifies parameters of prothrombin conversion and thrombin inactivation (22). Prothrombin conversion is quantified by the total amount of prothrombin conversion (PC_tot_) and the maximum prothrombin conversion rate (PC_max_). In previous studies, we have shown that prothrombin conversion is increased in conditions associated with an increased risk of thrombotic events, such as the antiphospholipid syndrome (26), and after strenuous exercise (27).

Thrombin inactivation during the TG process is quantified as the amount of thrombin-inhibitors complexes formed (23). Furthermore, the thrombin decay capacity can be determined independently of the TG curve, and is an estimate for the capability of the sample to inhibit generated thrombin (23). Thrombin inactivation is known to be reduced in conditions associated with a thrombotic risk, such as liver cirrhosis (24) and kidney failure (22).

In this study, we aim to analyze the ABC-based treatment-induced changes increase in TG peak height and ETP by applying thrombin dynamics analysis. We aim to investigate whether ABC-based treatment increases prothrombin conversion, hampers thrombin inactivation, or both, to provide an explanation for the higher TG potential compared to PLHIV on other types of combination antiretroviral therapy.

## 2 Methods

### 2.1 Sample collection

We previously described the population investigated in this (17). The study was approved by the local ethics committee (CMO Arnhem-Nijmegen, The Netherlands; NL425561.091.12, 2012/550). Virally suppressed PLHIV and uninfected controls were enrolled in the study after obtaining written informed consent. Participants were excluded if they had either an active hepatitis B or C infection, if they had signs of other acute infections or if they had received coumarin derivates or direct oral anticoagulants. Blood was collected into vacuum tubes (1 volume 0.109 mol/L trisodium citrate to 9 volumes blood; Greiner Bio-One). Platelet poor plasma (PPP) was prepared by centrifugation at 2840 g for 10 minutes and stored at -80°C.

### 2.2 Thrombin generation

Thrombin generation was measured in PPP using the CAT assay (Diagnostica Stago, Asnière-sur-Seine, France), as described in more detail previously (17). Thrombin generation was measured after a 5 pM tissue factor and 4 µM phospholipid trigger was added in the presence and absence of thrombomodulin to examine the function of the anticoagulant activated protein C pathway. TG parameters lag time, time-to-peak, peak, ETP and velocity index were calculated using the dedicated Thrombinoscope software (Diagnostica Stago, Asnière-sur-Seine, France). The lag time is defined as the time point at which the burst of TG starts, which is defined as 1/6^th^ of the peak height. The peak height represents the highest active thrombin concentration detectable. The time-to-peak is the time until the peak height is reached. The ETP is defined as the area under the curve and represents the total thrombin potential that a plasma sample can generate. The velocity index was calculated as peak height/(time-to-peak – lag time). The generated TG curves were used in thrombin dynamics analysis, as described below.

### 2.3 Thrombin dynamics

The TG curve is the net result of prothrombin conversion and thrombin inactivation, and the course of prothrombin conversion can therefore be calculated from a TG curve and its thrombin inactivation (22, 23). The prothrombin conversion curve is quantified by the area under the curve, which is defined as the total amount of prothrombin converted (PC_tot_) during the TG test, and the peak height of the prothrombin conversion curve, which is defined as the maximum rate of prothrombin conversion (PC_max_). Additionally, the amount of thrombin-antithrombin (T-AT) and thrombin-α_2_Macroglobulin (T-α_2_M) complexes formed during the experiment are quantified (23, 25, 26). The rate of thrombin inactivation was quantified by the thrombin decay constant (TDC), which is the pseudo-first order decay constant for thrombin inhibition by antithrombin, α_2_M and fibrinogen (22).

### 2.4 Coagulation and inflammatory factors

Fibrinogen levels were measured using the Clauss method on the STart (Diagnostica Stago, France). Antithrombin levels were measured chromogenically on the automated coagulation analyzer STA-R max using STA-Chrom ATIII reagents, according to manufacturer’s specifications (Diagnostica Stago, Asnière-sur-Seine, France) (28). Plasma α_2_M levels were measured with an in-house chromogenic assay as previously described by Kremers et al. (22).

Prothrombin levels were determined with an in-house sandwich ELISA assay (17) using a sheep anti-human prothrombin polyclonal antibody and a HRP-conjugated sheep anti-human prothrombin polyclonal antibody (Affinity Biologicals, Ancaster, Canada). Total VWF antigen levels were determined in an in-house developed sandwich ELISA using a polyclonal rabbit anti-VWF antibody and HRP-conjugated polyclonal rabbit anti-VWF antibody (Dako, Glostrup, Denmark). Active VWF levels were measured in an in-house ELISA as previously described in detail (29), using an anti-active VWF antibody (1.98 μg/ml) and a HRP-conjugated anti-VWF antibody. The determination of VWF propeptide levels was performed with an ELISA by a VWF propeptide antibody pair (CLB-PRO 35/CLB-PRO 14.3 - HRP) and VWF propeptide tool set for ELISA (Sanquin, Amsterdam, the Netherlands) as described previously (30). Optical densities (OD) were measured at 450 nm using an ELx808 Absorbance Microplate Reader (Biotek, Bad Friedrichshall, Germany).

### 2.5 Statistics

Statistical analysis was performed using IBM SPSS version 27 GraphPad Prism version 8. Normality of the data was assessed using the Shapiro-Wilk test. Data are presented as median with interquartile range (IQR). Comparison between groups was performed by either the Mann-Whitney test or Chi^2^ test. P-values <0.05 were considered statistically significant.

## 3 Results

Several hemostatic biomarkers, prothrombin conversion and thrombin inactivation were studied in 208 PLHIV on stable cART, and 56 controls without HIV. PLHIV were more often male (91.3% *vs.* 60.7%, p<0.001), and older (52 (46-59) *vs.* 30 (26-53) years, p<0.001) compared to uninfected controls (**Table 1**). As shown in **Table 1**, 94 PHLIV were treated with an ABC-containing regimen. Additionally, 114 participants were receiving a non-ABC containing regiment, most predominantly TDF (n=92). Previous thrombotic events such as myocardial infarction, stroke and venous thromboembolism were reported respectively in 10/208 (4.8%), 3/208 (1.4%) and 4/208 (1.9%) of PLHIV and did not differ significantly between PLHIV with or without ABC-use (**Table 1**).

**Table 1:** General characteristic, treatment strategy and comorbidities of people living with HIV (PLHIV), either on an abacavir (ABC) regimen or non-ABC regimen and controls without HIV.

	No ABC	ABC	Controls without HIV	ABC *vs.* No ABC	HC *vs.* PLHIV
General characteristic					
N	114	94	56		
Sex (female) (%)	13 (11.4)	5 (5.3)	22 (39.3)	0.192	<0.001
Age [median (IQR)]	53.0 (47.0, 60.0)	50.0 (41.5, 58.0)	30.0 (25.8, 53.0)	0.086	<0.001
BMI [median (IQR)]	24.2 (22.4, 26.0)	23.8 (21.8, 26.2)	23.8 (21.5, 25.6)	0.469	0.518
Treatment					
NNRTI [N (%)]	44 (38.6)	17 (18.1)		0.002	
PI [N (%)]	26 (22.8)	6 (6.4)		0.004	
INSTI [N (%)]	67 (58.8)	73 (77.7)		0.006	
NRTI backbone					
TDF [N (%)]	92 (80.7)	1 (1.1)		<0.001	
AZT [N (%)]	3 (2.6)	1 (1.1)		0.755	
FTC [N (%)]	91 (79.8)	0 (0.0)		<0.001	
3TC [N (%)]	14 (12.3)	91 (96.8)		<0.001	
Comorbidities					
Previous myocardial infarction [N (%)]	6 (5.3)	4 (4.3)		1.000	
Previous stroke [N (%)]	2 (1.8)	1 (1.1)		0.999	
Venous thromboembolism [N (%)]	3 (2.6)	1 (1.1)		0.755	
Smoking [N (%)]	33 (28.9)	24 (25.5)		0.694	
Hypercholesterolemia [N (%)]	33 (28.9)	23 (24.5)		0.570	
Hypertension [N (%)]	23 (20.2)	15 (16.0)		0.546	
Diabetes mellitus [N (%)]	6 (5.3)	2 (2.1)		0.419	
Family history of CVD [N (%)]	59 (51.8)	44 (46.8)		0.568	
Cholesterol-lowering drugs [N (%)]	35 (30.7)	21 (22.3)		0.232	
Antihypertensive drugs [N (%)]	27 (23.7)	20 (21.3)		0.805	
Acetyl salicylic acid [N (%)]	10 (8.8)	8 (8.5)		1.000	

Data are shown as median and interquartile range, or number and percentage. Statistical differences between groups were analyzed by the Mann-Whitney test, or the Chi-square test, depending on variable type. This cohort was previously described in relation to D-dimer and thrombin generation measurements (17).

NNRTI, non-nucleoside reverse transcriptase inhibitor; PI, protease inhibitor; INSTI, integrase inhibitor; NRTI, nucleoside/nucleotide reverse transcriptase inhibitor; TDF, tenofovir disoproxil fumarate; AZT, zidovudine; FTC, emtricitabine; 3TC, lamivudine.

**Figure 1:**
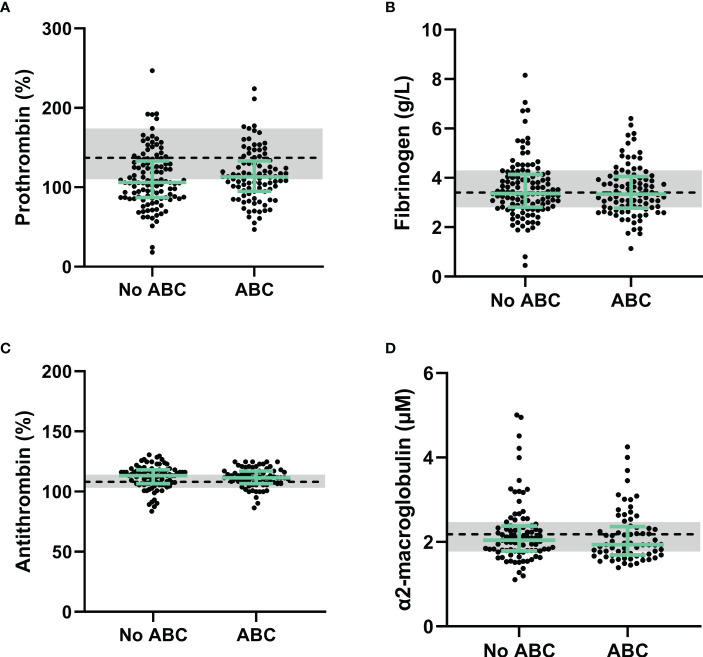
Procoagulant and anticoagulant factors in PLHIV stratified for ABC-based treatment. **(A)** Although prothrombin levels in PLHIV were lower than uninfected controls, prothrombin levels did not differ between PLHIV with an ABC-based treatment or a non-ABC-based treatment. **(B-D)** Fibrinogen **(B)**, antithrombin **(C)** and α2-macroglobulin **(D)** levels were comparable between ABC-based and non-ABC-based treated PLHIV, and between PLHIV and uninfected controls. Data of PLHIV participants with and without ABC-based treatment are represented as dots with green bars indicating the group median and interquartile range. For comparison, the median of the controls without HIV is shown as a black dashed line and the interquartile range is depicted as a grey box. ABC, abacavir; ETP, endogenous thrombin potential; PLHIV, people living with HIV;.

Lower prothrombin levels in PLHIV than controls without HIV could suggest a consumption of coagulation factors *in vivo*, especially in combination with elevated D-dimer levels (17). **Figure 1A** shows that prothrombin levels do not differ between PLHIV treated with ABC *vs.* other cART backbones. Additionally, fibrinogen levels were comparable between PLHIV on ABC-based or non-ABC-based treatment (**Figure 1B**). Moreover, plasma levels of natural anticoagulants antithrombin and α_2_-macroglobulin did not differ between PLHIV on ABC-based or non-ABC-based treatment, and between PLHIV and uninfected controls (**Figures 1C, D**).

**Figure 2:**
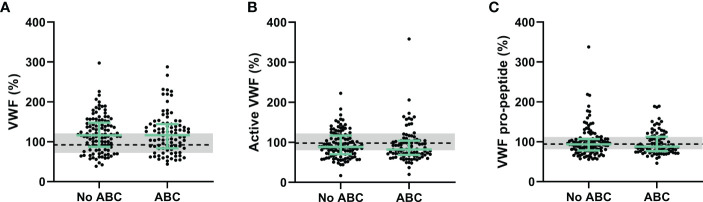
Von Willebrand factor as a biomarker of endothelial wall function in PLHIV, stratified for ABC treatment. **(A-C)** Von Willebrand Factor **(A)**, active Von Willebrand Factor **(B)** and Von Willebrand Factor pro-peptide **(C)** levels did not differ between PLHIV on ABC-based or non-ABC-based treatment, and between PLHIV and controls without HIV. Data of PLHIV with and without ABC-based treatment are represented as dots with green bars indicating the group median and interquartile range. For comparison, the median of the controls without HIV is shown as a black dashed line and the interquartile range is depicted as a grey box. ABC, abacavir; PLHIV, people living with HIV; VWF; Von Willebrand Factor.

Furthermore, endothelial damage could contribute to the prothrombotic phenotype as associated with ABC-based treatment in PLHIV. We quantified Von Willebrand factor (VWF) in its native and activated form, and the pro-peptide of VWF form as biomarkers of endothelial damage (**Figure 2**). VWF levels were significantly higher in PLHIV than uninfected controls (163 (118-204) *vs*. 129 (101-169), p =0.015) and active VWF were significantly lower in PLHIV (144 (115-183) *vs*. 164 (134-204), p=0.014). Nevertheless, VWF levels, active VWF levels and VWF pro-peptide levels did not differ significantly in PLHIV receiving ABC-based or non-ABC based treatment.

**Figure 3:**
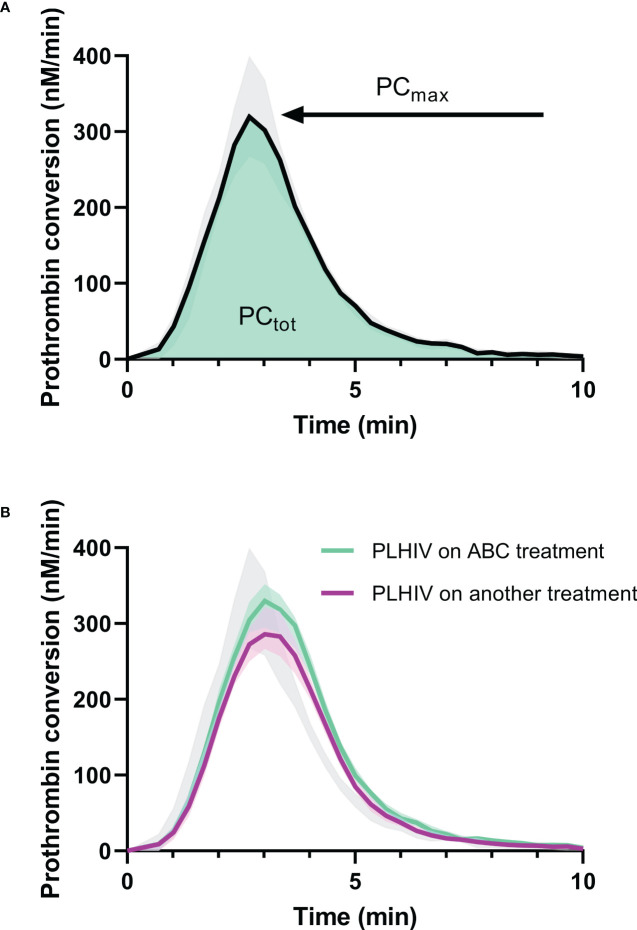
Prothrombin conversion curves in PLHIV and controls without HIV. **(A)** The median prothrombin conversion curves in controls without HIV with the 95% confidence interval marked as grey shading. The quantification of prothrombin conversion parameters PC_tot_ (area-under-the-curve, green) and PC_max_ (peak height of the curve) are indicated. **(B)** The median prothrombin conversion curves in PLHIV on ABC treatment (green) and PLHIV on other treatment regimens (purple), each with their 95% confidence interval depicted as shading. The 95% confidence interval of the controls without HIV is indicated in grey for comparison.

**Figure 4:**
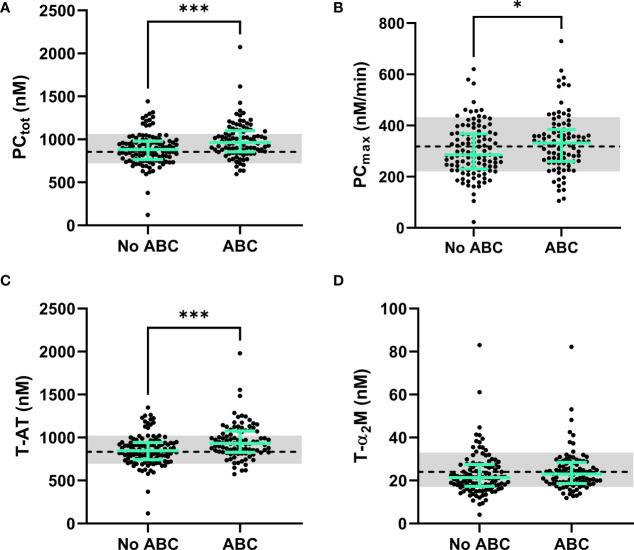
The dynamics of thrombin generation in PLHIV, stratified for ABC treatment. **(A)** The total amount of prothrombin converted was significantly higher in PLHIV on ABC treatment compared to other treatment regimes. **(B)** The maximum rate of prothrombin activation by the prothrombinase complex showed significantly higher rates in ABC treated individuals. **(C)** Thrombin-antithrombin complex formation was significantly higher in ABC treated participants compared to participants on other treatment regimes. **(D)** Thrombin-α_2_-macroglobulin complex formation did not differ between ABC treated participants and participants on other treatment regimes. Data of PLHIV participants with and without ABC treatment are represented as dots with green bars indicating the group median and interquartile range. For comparison, the median of the controls without HIV is shown as a black dashed line and the interquartile range is depicted as a grey box. ***P<0.001 and *p<0.05 according to the Mann-Whitney test.

We previously described an increase of thrombin generation in PLHIV on ABC-based treatment in the same cohort (17). The thrombin dynamics method was used to quantify the kinetics of prothrombin conversion, i.e. the production of thrombin, and the inactivation of active thrombin (**Figures 3**, **4**).

Prothrombin conversion and thrombin inactivation parameters were quantified in PLHIV on ABC-based treatment, and non-ABC-based treatments to find an explanation for the increased thrombin generation previously found in PLHIV on ABC-based treatment. The total amount of prothrombin converted was quantified as the area under the curve of each prothrombin conversion curve (PC_tot_) and the peak height of the prothrombin conversion curve was quantified as the maximum rate of prothrombin conversion (PC_max_) throughout the measurement (**Figure 3A**). **Figure 3B** shows that the mean peak height of the prothrombin conversion curve is higher in ABC-treated PLHIV compared to PLHIV on other treatment regimes.

The individual quantification of prothrombin conversion parameters is shown in **Figure 4**. The total amount of prothrombin converted (PC_tot;_ **Figure 4A**; 990 nM ± 187 nM *vs.* 990 nM ± 213 nM; p=0.0004) and maximum rate of prothrombin conversion (PC_max_; **Figure 4B**; 334 nM/min ± 112 nM/min *vs.* 303 nM/min ± 101 nM/min; p=0.0487) were significantly higher in PLHIV on ABC-based treatment compared to other treatments (**Figure 4B**). The increase in prothrombin conversion in PLHIV on ABC-based treatment resulted in a significantly higher amount of thrombin-antithrombin (T-AT) complexes formed PLHIV on ABC-based treatment (**Figure 4C**; 958 nM ± 208 nM *vs.* 858 nM ± 180 nM; p=0.0003), which is a main inhibitor of thrombin. The formation of thrombin-α_2_-macroglobulin (T-α_2_M) complexes did not differ between participants with ABC-based and non-ABC-based treatments (**Figure 4D**).

**Figure 5:**
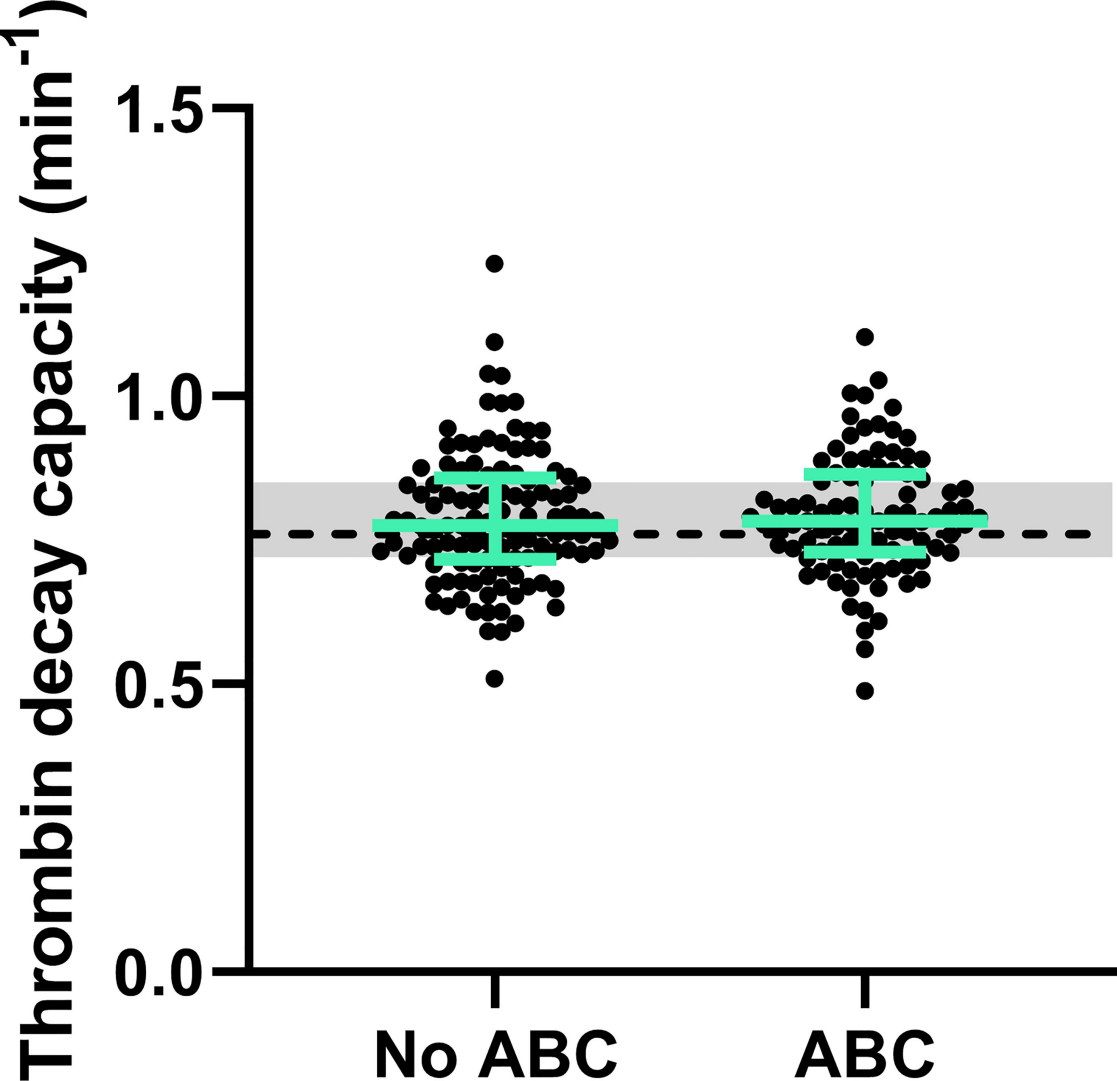
Natural anticoagulant activity in PLHIV, stratified for ABC treatment. The thrombin decay capacity does not differ between PLHIV treated with ABC or another treatment regime. The data of PLHIV participants with and without ABC treatment is represented as dots with green bars indicating the group median and interquartile range. For comparison, the median of the controls without HIV is shown as a black dashed line and the interquartile range is depicted as a grey box.

Moreover, the thrombin decay capacity was determined based on the levels of the natural anticoagulants and the modulating effect of fibrinogen (**Figure 5**), and was comparable both between PLHIV and individuals without, regardless of the chosen treatment strategy.

## 4 Discussion

Debate remains on whether specific antiretroviral drugs, such as ABC, increase the risk for CVD, including stroke and myocardial infarction (3–6, 31). In the present study, we performed an in-depth analysis of the mechanistical changes underlying the increased thrombin generation in PLHIV on ABC by applying thrombin dynamics analysis (22, 23). The total amount of prothrombin converted and the maximum rate at which prothrombin could be converted were higher PLHIV on ABC-based treatment compared to PLHIV on other treatments. Increased prothrombin conversion has been shown to be associated with a prothrombotic phenotype in several populations with an increased risk of thrombosis, including antiphospholipid syndrome patients (26), liver cirrhosis patients (24) and patients infected with COVID-19 (32). This is more pronounced when the anticoagulant pathway is not increased or even decreased, shifting the balance towards thrombosis (23). Moreover, a reduction of thrombin inactivation itself has been shown to be associated with an increased risk of thrombosis in COVID-19 patients (32). Even the rebalanced prothrombin conversion and thrombin inactivation processes in liver cirrhosis, in which the thrombin generation curve appears to be normal, is associated with a higher risk of thrombotic events (24).

In this cohort of PLHIV, thrombin inactivation, as quantified by the thrombin decay capacity, did not differ between uninfected controls and PLHIV, irrespective of their treatment regime. Together, these findings indicate that the balance between pro- and anticoagulant processes shifts towards the procoagulant prothrombin conversion process, leading to the increase of thrombin generation in PLHIV on ABC-based treatment, as thrombin inactivation was unaltered. The elevation of prothrombin conversion could explain the increased risk of thrombotic events associated with ABC (33, 34). Whether this is a direct or an indirect consequence of ABC treatment, cannot be concluded from this study. However, *in vitro* addition of ABC or its active metabolite carbovir diphosphate to platelet rich plasma does not cause and increase of thrombin generation (35). This suggests that the effect of ABC on thrombin generation and the risk of thrombosis might be indirect.

The cross-sectional design of the study prevents the investigation of causal interference of thrombin generation and thrombin dynamics parameters and thrombotic risk in PLHIV, as the primary aim of the study was to investigate the association between thrombin dynamics and cART. A limitation of the study is that we were unable to study these associations in platelet rich plasma or whole blood, to include the effect of platelets. However, it has been previously reported that ABC treatment has no prothrombotic effect on platelets *in vitro* (35). Another limitation is the difference in demographics between the controls without HIV and PLHIV. Nevertheless, the cohort contained an existing control group with matching demographics consisting of PLHIV in non-ABC-based treatments.

In conclusion, we found that the use of ABC is associated with increased prothrombin conversion without changes in thrombin inactivation. The net result of these changes is an increase in the TG potential. This finding explains the reported increase in thrombin generation ETP in PLHIV on ABC-based treatment compared to PLHIV on non-ABC-based treatments. Moreover, this increase in prothrombin conversion and subsequently ETP may contribute to the increased risk of thrombotic events PLHIV receiving ABC-based treatment.

## Data availability statement

The original contributions presented in the study are included in the article/**Supplementary Material**. Further inquiries can be directed to the corresponding author.

## Ethics statement

The studies involving human participants were reviewed and approved by CMO Arnhem-Nijmegen. The patients/participants provided their written informed consent to participate in this study.

## Author contributions

QY performed experiments, analyzed the data, and drafted the manuscript. SH performed experiments, analyzed the data, and drafted the manuscript. WH collected the samples, included the participants and co-wrote the manuscript. LW collected the samples, included the participants and co-wrote the manuscript. MN supervised the data collection and co-wrote the manuscript. RL-K performed thrombin dynamics analysis, performed analyses, supervised the data collection, and co-wrote the manuscript. AV designed the study, supervised the project and co-wrote the manuscript. BL designed the study, supervised the project, and co-wrote the manuscript. QM designed the study, supervised the project, and co-wrote the manuscript. All authors contributed to the article and approved the submitted version.
