# Supplementary material for: Abacavir use is associated with increased prothrombin conversion
Source: Front Immunol. 2023 Apr 14;14:1182942. doi: 10.3389/fimmu.2023.1182942 (PMC10140416; doi:10.3389/fimmu.2023.1182942)
Supplement: Supplementary file 1 [file DataSheet_1.docx]

Supplementary Material

**ABACAVIR USE IS ASSOCIATED WITH INCREASED PROTHROMBIN CONVERSION**

**Qiuting Yan1,2†, Shengshi Huang1,2†, Wouter van der Heijden3, Marisa Ninivaggi1, Lisa van de Wijer3, Romy de Laat-Kremers1, Andre J. Van der Ven3, Bas de Laat1,4¶*, Quirijn de Mast3¶**

*** Correspondence:** Corresponding Author: b.delaat@thrombin.com

**Supplementary figure 1: Thrombin generation in a cohort of people living with HIV (PLHIV), stratified for ABC-based treatment.** (A) The lag time did not differ between PLHIV using ABC-based or non-ABC-based treatment. (B) The peak height was significantly higher in subjects using ABC-based treatment. (C) The time-to-peak did not differ in PLHIV on ABC-based and ABC-based treatment. (D) The ETP was significantly higher in subjects undergoing an ABC-based treatment. (E) The velocity index did not differ significantly between PLHIV on an ABC-based or non-ABC-based treatment. (F) The inhibitory actions of thrombomodulin on the thrombin generation ETP did not differ between subjects using ABC-based or non-ABC-based treatment. The data is represented as dots with green bars indicating the group median and interquartile range. Peak height, ETP, and velocity index were shown as a percentage of each variable in pooled normal plasma. ***P<0.001 and *p<0.05 according to the Mann-Whitney test. ABC, abacavir; ETP, endogenous thrombin potential; PLHIV, people living with HIV;
